# Supplementary material for: Molecular Detection of Antimalarial Drug Resistance in Plasmodium vivax from Returned Travellers to NSW, Australia during 2008–2018
Source: Pathogens. 2020 Feb 5;9(2):101. doi: 10.3390/pathogens9020101 (PMC7168284; doi:10.3390/pathogens9020101)
Supplement: Supplementary file 1 [file pathogens-09-00101-s001.pdf]

Supplementary Materials

Table S1: The number of successful sequences obtained from 120 clinical samples.

**Countries**	**Target genes**			
	Pvmdr1	***Pvcrt-o***	***Pvdhfr***	***Pvdhps***
India	51	48	50	54
PNG	29	20	33	34
Pakistan	17	10	17	17
Indonesia	5	4	5	5
Thailand	2	2	2	2
Solomon	2	2	2	2
South Korea	1	0	1	1
Cambodia	1	1	1	0
SEAN	1	1	1	1
**Total**	**109**	**88**	**112**	**116**

Pvmdr1 = *P. vivax* multidrug resistance gene
Pvcrt-o = *P. vivax* putative transporter protein gene
Pvdhfr = *P. vivax* dihydrofolate reductase gene
Pvdhps = *P. vivax* dihydropteroate synthase gene

Table S2: Total of targeted sequences obtained from clinical samples.

ID	Countries	Year	*Pvmdr1*		*Pvcrt-o*	*Pvdhfr*						*Pvdhps*				
			Y976**F**	F1076**L**	**K**10 insertion	F57**L/I**	S58**R**	T61**M**	S117**T/N**	I173**L**/**F**	Repeat variants*	S382**A**	A383**G**	K512**M/E**	A553**G**	V585**A**
V235	Pakistan	2008	**F**	**L**	WT	F	**R**	T	**N**	I	Type2	S	**G**	K	A	V
V255	PNG*	2008	**F**	**L**	WT	**L**	**R**	**M**	**T**	I	Type3	S	A	K	A	V
V247	PNG*	2008	Y	**L**	WT	**L**	**R**	**M**	**T**	I	Type3	S	A	K	A	V
V250	PNG*	2008	Y	**L**	WT	**L**	**R**	**M**	**T**	I	Type3	S	A	K	A	V
V298	Indonesia	2008	Y	**L**	WT	**L**	**R**	**M**	**T**	I	Type3	S	A	K	A	V
V299	India	2008	Y	**L**	WT	F	**R**	T	**N**	I	Type1	S	A	K	A	V
V225	India	2008	Y	**L**	WT	F	S	T	S	I	Type2	S	A	K	A	V
V243	India	2008	Y	**L**	WT	F	S	T	S	I	Type1	S	A	K	A	V
V249	India	2008	Y	**L**	WT	F	S	T	S	I	Type1	S	A	K	A	V
V296	India	2008	Y	**L**	WT	F	S	T	S	I	Type1	S	A	K	A	V
V303	PNG*	2009	**F**	**L**	WT	**L**	**R**	**M**	**T**	I	Type3	S	A	K	A	V
V183	India	2009	Y	**L**	WT	**L**	**R**	**M**	**T**	I	Type3	S	A	K	A	V
V301	PNG*	2009	Y	**L**	WT	**L**	**R**	**M**	**T**	I	Type3	S	A	K	A	V
V202	PNG*	2009	Y	**L**	WT	F	S	T	**N**	I	Type1	S	A	K	A	V
V197	India	2009	Y	**L**	WT	F	S	T	S	I	Type1	S	A	K	A	V
V302	India	2009	Y	**L**	WT	F	S	T	S	I	Type1	S	A	K	A	V
V163	Thailand	2010	Y	**L**	WT	F	**R**	T	**N**	I	Type2	S	**G**	K	**G**	V
V174	India	2010	Y	**L**	WT	F	**R**	T	**N**	I	Type1	S	**G**	K	**G**	V
V162	PNG*	2010	**F**	**L**	WT	**L**	**R**	**M**	**T**	I	Type3	S	A	K	A	V
V180	India	2010	Y	**L**	WT	F	**R**	T	**N**	I	Type1	S	A	K	A	V
V177	India	2010	Y	**L**	WT	F	S	T	S	I	Type2	S	A	K	A	V
V156	PNG*	2010			WT	**L**	**R**	**M**	**T**	I	Type3	S	A	K	A	V
V158	PNG*	2010	**F**	**L**		F	**R**	T	**N**	I	Type1	S	A	K	A	V

V167	India	2010	Y	**L**		F	**R**	T	**N**	I	Type1	S	A	K	A	V
V165	PNG*	2010	**F**	**L**								S	A	K	A	V
V132	PNG*	2011	**F**	**L**	WT	**L**	**R**	**M**	**T**	I	Type3	S	A	K	A	V
V340	PNG*	2011	**F**	**L**	WT	**L**	**R**	**M**	**T**	I	Type3	S	A	K	A	V
V130	Solomon*	2011	**F**	**L**	WT	**L**	**R**	**M**	**T**	I	Type3	S	A	K	A	V
V335	PNG*	2011	**F**	**L**	WT	**L**	**R**	T	S	I	Type1	S	A	K	A	V
V330	PNG*	2011	Y	**L**	WT	**L**	**R**	T	S	I	Type1	S	A	K	A	V
V147	Indonesia	2011	**F**	**L**	WT	F	S	T	S	I	Type1	S	A	K	A	V
V331	PNG*	2011	**F**	**L**	WT	F	S	T	S	I	Type1	S	A	K	A	V
V329	PNG*	2011	Y	**L**	WT	F	S	T	S	I	Type1	S	A	K	A	V
V341	PNG*	2011			WT	F	S	T	S	I	Type1	S	A	K	A	V
V322	PNG*	2011	**F**	**L**		**L**	**R**	T	S	I	Type1	S	A	K	A	V
V324	PNG*	2011	**F**	**L**		**L**	**R**	**M**	**T**	I	Type3	S	A	K	A	V
V325	PNG*	2011	**F**	**L**		**L**	**R**	**M**	**T**	I	Type3	S	A	K	A	V
V327	PNG*	2011	**F**	**L**		**L**	**R**	**M**	**T**	I	Type3	S	A	K	A	V
V336	PNG*	2011	**F**	**L**		F	S	T	S	I	Type1	S	A	K	A	V
V338	PNG*	2011	**F**	**L**		F	S	T	S	I	Type1	S	A	K	A	V
V134	Cambodia	2011	Y	**L**	**K**	F	**R**	T	**N**	I	Type2					
V343	PNG*	2011				F	S	T	S	I	Type1	S	A	K	A	V
V339	PNG*	2011				F	S	T	S	I	Type1	S	A	K	A	V
V323	PNG*	2011				**L**	**R**	T	S	I	Type1	S	A	K	A	V
V320	PNG*	2011				**L**	**R**	**M**	**T**	I	Type3	S	A	K	A	V
V328	PNG*	2011	**F**	**L**								S	A	K	A	V
V142	India	2011	Y	**L**								S	A	K	A	V
V117	Indonesia	2012	**F**	**L**	WT	F	S	T	S	I	Type1	S	**G**	K	A	V
V149	India	2012	Y	**L**	WT	F	**R**	T	**N**	I	Type1	S	A	K	A	V
V126	India	2012	Y	**L**	WT	F	S	T	**N**	I	Type1	S	A	K	A	V
V119	Pakistan	2012	Y	F	WT	F	**R**	T	**N**	I	Type1	S	A	K	A	V
V151	India	2012	Y	**L**	WT	F	S	T	S	I	Type1	S	A	K	A	V

V127	Pakistan	2012	Y	**L**	WT	F	S	T	S	I	Type1	S	A	K	A	V
V150	Pakistan	2012	Y	**L**	WT	F	S	T	S	I	Type3	S	A	K	A	V
V128	Pakistan	2012	Y	F	WT	F	S	T	S	I	Type2	S	A	K	A	V
V102	India	2013	**F**	**L**	WT	**I**	**R**	**M**	**T**	I	Type1	S	**G**	K	**G**	V
V93	India	2013	Y	**L**	**K**	F	S	T	S	I	Type1	S	A	K	A	V
V97	India	2013	Y	**L**	**K**	F	S	T	S	I	Type1	S	A	K	A	V
V103	India	2013	Y	**L**	WT	F	S	T	S	I	Type2	S	**G**	K	A	V
V95	India	2013	Y	**L**	WT	F	S	T	S	I	Type1	S	A	K	A	V
V96	India	2013	Y	**L**	WT	F	S	T	S	I	Type1	S	A	K	A	V
V98	India	2013	Y	**L**	WT	F	S	T	S	I	Type1	S	A	K	A	V
V105	India	2013	Y	**L**	WT	F	S	T	S	I	Type1	S	A	K	A	V
V106	Pakistan	2013	Y	**L**	WT	F	S	T	S	I	Type1	S	A	K	A	V
V101	Thailand	2013	Y	**L**	WT	F	S	T	S	I	Type1	S	A	K	A	V
V99	India	2013			**K**	F	**R**	T	**N**	I	Type1	S	**G**	K	**G**	V
V114	India	2013	Y	**L**		F	S	T	S	I	Type1	S	A	K	A	V
V110	India	2013	Y	F												
V70	SEAN*	2014	**F**	**L**	**K**	**L**	**R**	**M**	**T**	I	Type1	S	**G**	K	**G**	V
V64	India	2014	Y	F	**K**	F	**R**	T	**N**	I	Type1	S	**G**	K	**G**	V
V65	Indonesia	2014	**F**	**L**	WT	**L**	**R**	**M**	**T**	I	Type1	S	A	K	A	V
V59	India	2014	Y	F	WT	F	**R**	T	**N**	I	Type1	S	**G**	K	**G**	V
V60	India	2014	Y	F	WT	F	**R**	T	**N**	I	Type1	S	**G**	K	**G**	V
V73	Solomon*	2014	Y	**L**	WT	**L**	**R**	**M**	**T**	I	Type3	S	A	K	A	V
V67	Pakistan	2014	Y	**L**	WT	F	**R**	T	**N**	I	Type1	S	A	K	A	V
V68	Pakistan	2014	Y	**L**	WT	F	**R**	T	**N**	I	Type1	S	A	K	A	V
V72	India	2014	Y	**L**	WT	**L**	**R**	T	S	I	Type2	S	A	K	A	V
V63	India	2014	Y	F	**K**	F	S	T	S	I	Type1	S	A	K	A	V
V74	India	2014			WT	F	**R**	T	**N**	I	Type1	S	A	K	A	V
V61	India	2014	Y	**L**	WT							S	A	K	A	V
V40	PNG*	2015	**F**	**L**	WT	**L**	**R**	**M**	**T**	I	Type3	S	A	K	A	V

V49	India	2015	Y	**L**	**K**	F	**R**	T	**N**	I	Type1	S	A	K	A	V
V48	Pakistan	2015	Y	**L**	WT	F	S	T	**N**	I	Type1	S	A	K	A	V
V47	Pakistan	2015	Y	**L**	WT	F	S	T	S	I	Type1	S	A	K	A	V
V42	Indonesia	2015	**F**	**L**		**L**	**R**	**M**	**T**	I	Type1	S	A	K	**G**	V
V43	India	2015	Y	**L**								S	A	K	A	V
V50	India	2015				F	**R**	T	**N**	I	Type1					
V20	PNG*	2016	**F**	**L**	WT	**L**	**R**	**M**	**T**	I	Type3	S	A	K	A	V
V25	India	2016	Y	**L**	WT	F	S	T	S	I	Type1	S	A	K	**G**	V
V29	India	2016	Y	F	WT	**L**	**R**	**M**	**T**	I	Type3	S	A	K	A	V
V24	India	2016	Y	**L**	WT	F	S	T	S	I	Type1	S	A	K	A	V
V31	India	2016	Y	**L**	WT	F	S	T	S	I	Type1	S	A	K	A	V
V32	India	2016			WT	F	S	T	S	I	Type1	S	A	K	A	V
V34	India	2016	Y	**L**	WT							S	A	K	A	V
V3	PNG*	2017	**F**	**L**	WT	**L**	**R**	**M**	**T**	I	Type1	S	**G**	K	**G**	V
V11	India	2017	**F**	F	**K**	F	**R**	T	**N**	I	Type1	S	A	K	A	V
V5	India	2017	**F**	F	**K**	F	S	T	S	I	Type1	S	A	K	A	V
V6	India	2017	**F**	F	**K**	F	S	T	S	I	Type1	S	A	K	A	V
V18	PNG*	2017	Y	**L**	WT	**L**	**R**	T	S	I	Type1	S	A	K	A	V
V1	India	2017	**F**	F	WT	F	S	T	S	I	Type1	S	A	K	A	V
V7	India	2017	**F**	F	WT	F	S	T	S	I	Type1	S	A	K	A	V
V8	India	2017	**F**	F	WT	F	S	T	S	I	Type1	S	A	K	A	V
V9	India	2017	**F**	F	WT	F	S	T	S	I	Type1	S	A	K	A	V
V14	India	2017	Y	**L**	WT	F	S	T	S	I	Type1	S	A	K	A	V
V15	India	2017	Y	**L**	WT	F	S	T	S	I	Type1	S	A	K	A	V
V13	India	2017			WT	F	S	T	**N**	I	Type1	S	A	K	A	V
V16	Pakistan	2017	Y	**L**		F	**R**	T	**N**	I	Type1	S	A	K	A	V
V12	PNG*	2017	**F**	**L**		**L**	**R**	**M**	**T**	I	Type3	S	A	K	A	V
V19	PNG*	2017	**F**	**L**	WT	**L**	**R**	**M**	**T**	I	Type1					
V314	India	2018	Y	**L**	WT	F	S	T	S	I	Type1	S	A	K	A	V

V310	Pakistan	2018	Y	**<u>L</u>**		F	S	T	S	I	Type1	S	A	K	A	V
V312	Pakistan	2018	Y	**<u>L</u>**		F	S	T	S	I	Type1	S	A	K	A	V
V313	Pakistan	2018	Y	**<u>L</u>**		F	S	T	S	I	Type3	S	A	K	A	V
V315	India	2018	Y	**<u>L</u>**		F	S	T	S	I	Type2	S	A	K	A	V
V316	South Korea	2018	Y	**<u>L</u>**		F	S	T	N	I	Type2	S	A	K	A	V
V317	PNG*	2018	**<u>F</u>**	**<u>L</u>**		**<u>L</u>**	**<u>R</u>**	**<u>M</u>**	**<u>T</u>**	I	Type3	S	A	K	A	V
V318	Pakistan	2018	Y	**<u>L</u>**		F	S	T	S	I	Type1	S	A	K	A	V
V319	Pakistan	2018	Y	**<u>L</u>**		F	S	T	S	I	Type2	S	A	K	A	V
V321	Pakistan	2018	Y	**<u>L</u>**		F	S	T	S	I	Type2	S	A	K	A	V

SEAN = Southeast Asian Nations
PNG = Papua New Guinea
Solomon = Solomon Islands
Pvmdr1 = *P. vivax* multidrug resistance gene
Pvcrt-o = *P. vivax* putative transporter protein gene
Pvdhfr = *P. vivax* dihydrofolate reductase gene
Pvdhps = *P. vivax* dihydropteroate synthase gene
*The nomenclature of the repeat variants followed that of Tantiamornkul et al. 2018.

Table S3: The table of mutations in four genes found in Indian samples between 2008 and 2018

ID	Year	*Pvmdr1*		*Pvcrt-o*	*Pvdhfr*					*Pvdhps*				
		Y976**F**	F1076**L**	**K**10 insertion	F57**L/I**	S58**R**	T61**M**	S117**T/N**	I173**L/F**	S382**A**	A383**G**	K512**M/E**	A553**G**	V585**A**
V299	2008	Y	**L**	WT	F	**R**	T	**N**	I	S	A	K	A	V
V225	2008	Y	**L**	WT	F	S	T	S	I	S	A	K	A	V
V243	2008	Y	**L**	WT	F	S	T	S	I	S	A	K	A	V
V249	2008	Y	**L**	WT	F	S	T	S	I	S	A	K	A	V
V296	2008	Y	**L**	WT	F	S	T	S	I	S	A	K	A	V
V183	2009	Y	**L**	WT	**L**	**R**	**M**	**T**	I	S	A	K	A	V
V197	2009	Y	**L**	WT	F	S	T	S	I	S	A	K	A	V
V302	2009	Y	**L**	WT	F	S	T	S	I	S	A	K	A	V
V174	2010	Y	**L**	WT	F	**R**	T	**N**	I	S	**G**	K	**G**	V
V180	2010	Y	**L**	WT	F	**R**	T	**N**	I	S	A	K	A	V
V177	2010	Y	**L**	WT	F	S	T	S	I	S	A	K	A	V
V167	2010	Y	**L**		F	**R**	T	**N**	I	S	A	K	A	V
V142	2011	Y	**L**							S	A	K	A	V
V149	2012	Y	**L**	WT	F	**R**	T	**N**	I	S	A	K	A	V
V126	2012	Y	**L**	WT	F	S	T	**N**	I	S	A	K	A	V
V151	2012	Y	**L**	WT	F	S	T	S	I	S	A	K	A	V
V102	2013	**F**	**L**	WT	**I**	**R**	**M**	**T**	I	S	**G**	K	**G**	V
V93	2013	Y	**L**	**K**	F	S	T	S	I	S	A	K	A	V
V97	2013	Y	**L**	**K**	F	S	T	S	I	S	A	K	A	V
V103	2013	Y	**L**	WT	F	S	T	S	I	S	**G**	K	A	V
V95	2013	Y	**L**	WT	F	S	T	S	I	S	A	K	A	V
V96	2013	Y	**L**	WT	F	S	T	S	I	S	A	K	A	V
V98	2013	Y	**L**	WT	F	S	T	S	I	S	A	K	A	V

V105	2013	Y	**L**	WT	F	S	T	S	I	S	A	K	A	V
V99	2013			**K**	F	**R**	T	**N**	I	S	**G**	K	**G**	V
V114	2013	Y	**L**		F	S	T	S	I	S	A	K	A	V
V110	2013	Y	F											
V64	2014	Y	F	**K**	F	**R**	T	**N**	I	S	**G**	K	**G**	V
V59	2014	Y	F	WT	F	**R**	T	**N**	I	S	**G**	K	**G**	V
V60	2014	Y	F	WT	F	**R**	T	**N**	I	S	**G**	K	**G**	V
V72	2014	Y	**L**	WT	**L**	**R**	T	S	I	S	A	K	A	V
V63	2014	Y	F	**K**	F	S	T	S	I	S	A	K	A	V
V74	2014			WT	F	**R**	T	**N**	I	S	A	K	A	V
V61	2014	Y	**L**	WT						S	A	K	A	V
V49	2015	Y	**L**	**K**	F	**R**	T	**N**	I	S	A	K	A	V
V43	2015	Y	**L**							S	A	K	A	V
V50	2015				F	**R**	T	**N**	I					
V25	2016	Y	**L**	WT	F	S	T	S	I	S	A	K	**G**	V
V29	2016	Y	F	WT	**L**	**R**	**M**	**T**	I	S	A	K	A	V
V24	2016	Y	**L**	WT	F	S	T	S	I	S	A	K	A	V
V31	2016	Y	**L**	WT	F	S	T	S	I	S	A	K	A	V
V32	2016			WT	F	S	T	S	I	S	A	K	A	V
V34	2016	Y	**L**	WT						S	A	K	A	V
V11	2017	**F**	F	**K**	F	**R**	T	**N**	I	S	A	K	A	V
V5	2017	**F**	F	**K**	F	S	T	S	I	S	A	K	A	V
V6	2017	**F**	F	**K**	F	S	T	S	I	S	A	K	A	V
V1	2017	**F**	F	WT	F	S	T	S	I	S	A	K	A	V
V7	2017	**F**	F	WT	F	S	T	S	I	S	A	K	A	V
V8	2017	**F**	F	WT	F	S	T	S	I	S	A	K	A	V
V9	2017	**F**	F	WT	F	S	T	S	I	S	A	K	A	V
V14	2017	Y	**L**	WT	F	S	T	S	I	S	A	K	A	V
V15	2017	Y	**L**	WT	F	S	T	S	I	S	A	K	A	V

V13	2017			WT	F	S	T	**N**	I	S	A	K	A	V
V314	2018	Y	**L**	WT	F	S	T	S	I	S	A	K	A	V
V315	2018	Y	**L**		F	S	T	S	I	S	A	K	A	V

Pvmdr1 = *P. vivax* multidrug resistance gene
Pvcrt-o = *P. vivax* putative transporter protein gene
Pvdhfr = *P. vivax* dihydrofolate reductase gene
Pvdhps = *P. vivax* dihydropteroate synthase gene

Table S4: The table of orthologous genes associated with drug resistance between *P. falciparum* and *P. vivax*

Plasmodium falciparum		*Plasmodium vivax*		Reference
Genes	Point Mutations	Orthologous Genes	Orthologous Point Mutations	
Pfmdr		*Pvmdr*		Suwanarusk et al 2007
Pfcrt		*Pvcrt*		Suwanarusk et al 2007
Pfdhfr	59<br>108<br>164	*Pvdhfr*	58<br>117<br>173	Hawkins et al 2007
Pfdhps	437<br>540<br>581	*Pvdhps*	383<br>512<br>553	Hawkins et al 2007

Pfmdr gene; a multidrug resistance gene in *P. falciparum, Pfcrt* gene; a putative transporter protein gene in *P. falciparum, Pfdhfr* gene; dihydrofolate reductase gene in *P. falciparum, Pfdhps* gene; dihydropteroate synthetase gene in *P. falciparum, Pvmdr* gene; a multidrug resistance gene in *P. vivax, Pvcrt* gene; a putative transporter protein gene in *P. vivax, Pvdhfr* gene; dihydrofolate reductase gene in *P. vivax,* and *Pvdhps* gene; dihydropteroate synthetase gene in *P. vivax*
